# Metabolic syndrome in hypertensive women in the age of menopause: a case study on data from general practice electronic health records

**DOI:** 10.1186/s12911-018-0601-2

**Published:** 2018-04-02

**Authors:** Šefket Šabanović, Majnarić Trtica Ljiljana, František Babič, Michal Vadovský, Ján Paralič, Aleksandar Včev, Andreas Holzinger

**Affiliations:** 1 Department for Internal Medicine, Family Medicine and the History of Medicine, Faculty of Medicine, Josip Juraj Strossmayer University, Osijek, Croatia, Huttlerova 10b, 31 000 Osijek, Croatia; 2 Department of Cybernetics and Artificial Intelligence, Technical University of Košice, Faculty of Electrical Engineering and Informatics, Letná 9/B, 042 00 Košice, Slovakia; 3 Medical University Graz, Institute for Medical Informatics/Statistic, Auenbruggerplatz 2/V, 8036 Graz, Austria

**Keywords:** General practice, Research, Routine data, Electronic health records, Computer methods for data anlysis, Menopausal women, Hypertension, Metabolic syndrome

## Abstract

**Background:**

There is potential for medical research on the basis of routine data used from general practice electronic health records (GP eHRs), even in areas where there is no common GP research platform. We present a case study on menopausal women with hypertension and metabolic syndrome (MS). The aims were to explore the appropriateness of the standard definition of MS to apply to this specific, narrowly defined population group and to improve recognition of women at high CV risk.

**Methods:**

We investigated the possible uses offered by available data from GP eHRs, completed with patients interview, in goal of the study, using a combination of methods. For the sample of 202 hypertensive women, 47–59 years old, a data set was performed, consisted of a total number of 62 parameters, 50 parameters used from GP eHRs. It was analysed by using a mixture of methods: analysis of differences, cutoff values, graphical presentations, logistic regression and decision trees.

**Results:**

The age range found to best match the emergency of MS was 51–55 years. Deviations from the definition of MS were identified: a larger cut-off value of the waist circumference measure (89 vs 80 cm) and parameters BMI and total serum cholesterol perform better as components of MS than the standard parameters waist circumference and HDL-cholesterol. The threshold value of BMI at which it is expected that most of hypertensive menopausal women have MS, was found to be 25.5. The other best means for recognision of women with MS include triglycerides above the threshold of 1.7 mmol/L and information on statins use. Prevention of CVD should focus on women with a new onset diabetes and comorbidities of a long-term hypertension with anxiety/depression.

**Conclusions:**

The added value of this study goes beyond the current paradigm on MS. Results indicate characteristics of MS in a narrowly defined, specific population group. A comprehensive view has been enabled by using heterogenoeus data and a smart combination of various methods for data analysis. The paper shows the feasibility of this research approach in routine practice, to make use of data which would otherwise not be used for research.

## Background

Strongly founded and evidence-based primary care (PC) is known to significantly improve health of the nations and the efficacy of use of health care (HC) resources [1, 2]. For its position at the interface between population and the HC system, general practice (GP) is the key PC discipline [3]. It is considered that efforts aimed to improve efficiency of GP within the HC system should be taken through strengthening the research capacity of this discipline [4]. This is because GP is a specific discipline, different from specialist medicine, and requires its own knowledge base to improve decision making [4, 5].

Decision making in GP often deals with uncertainty, as many patients present with early symptoms and signs of a disease that do not yet meet criteria for a diagnosis [6]. Older population, that makes the prominent part of GP patients, is usually characterised with multimorbidity (the coexistence of two or more chronic conditions) [7]. These patients are known to have complex HC needs that require solutions that go beyond the disease-based approaches of the traditional medicine, for which also evidence-based medicine (EBM) does not provide adequate answers [8]. This is one of the reasons why EBM, that draws primarily on randomised controlled trials and properly selected populations from tertiary care centres, is difficult to translate to the GP setting [9]. Rather, it is considered that research in GP has to be driven by problems and questions that are derived from its own practice [5].

The first attempt to build the science base of GP at large scale dated back to the end of the past century. To enable a wide access to data in GP, the research elities of this discipline initiated development of practice based research networks across Europe and wider [10]. With the advent of Information and Communication Technology (ICT) and electronic Health Records (eHRs) in PC, this initiative found new opportunities for realisation [11]. In many European countries, individual GP practices have been networked at the national level and episodes of care aggregated in a longitudinal way, to allow the common virtual platform for research [12, 13]. Experience from these countries have helped us learn on how to overcome barriers, while making the best of using the routinelly collected data from GP eHRs for research. It was showed that even from these databases, the number of research questions possible to be investigated is limited, including mostly pharmacoepidemiological and drug-safety issues and health service research [14, 15]. The key barriers, identified to date, include: a limited scope of data recorded in eHRs, non systematically recorded data on socio-economic and lifestyle factors, lack of compatibility in morbidity coding and prescribing guidelines, non uniformity in terminology and content meanings and the lack of links with other HC sectors and databases [16, 17].

In countries where there is no a “gatekeeping “role of GP, but people have the direct access to specialists, the problem is also in non systematically recorded data in GP eHRs [18]. On the contrary, some recent examples, based on integration of GP databases with other national registries, have emphasised the emerging opportunities that the “big data “analytical approaches could have in improving the quality of care and patients outcomes [19, 20]. This would be in a great part possible through using GP eHRS for identification of phenotypes, necessary for predictive modeling [21]. It is considered that opportunities for research that create upon GP databases could be practically endless if data of different types were combined together, including not only structured data (coded and numerical data), that are the easiest for computing, but also text narratives and images, and if different Machine Learning (ML) and other computer methods for complex data analysis were used in the process of problem solving [22].

### Motivation for this study

Motivation for this study came from our previous work, where we used multicomponent data sets, composed mostly of data from GP eHRs, and a combination of statistical and data mining methods, for comprehensive analysis of a research question [23–26]. This way, we could answer some important questions associated with uncertainty and complexity in decision making.

Through experience of this work, we came to the conclusion that in GP it is possible to perform a single-site study, without the need of using the common research database, if only structured data (diagnoses, list of medications, numerical data, etc.), known to be consistently recorded, are used for analysis and if the right question is asked of data. To enlarge the scope of data from GP eHRs, some other, but easy-to-obtain data sources have to be added to.

For some of our results we found confirmation in EMB. For some new findings, for which comprehensive analysis has allowed for, we found confirmation later on, in studies of other kind and other authors. Generalisation of these results is still important to achieve, possible through iteration and validation of the same study on other samples, by following the principles of the “bottom-up “research approach. Based on this experience, we believe that reasearch in GP can blow up, in spite of the current situation where the lack of the networked databases and the existence of some unresolved barriers pose limitations to the global use of GP eHRs, for nationwide and cross-country research.

### The case study

To illustrate the research approach that we recommend for use in GP, we used the case study on menopausal women with hypertension. This is a complex issue for which, however, the most of data are available in GP eHRs. There were several other reasons to support this choice.

Middle-aged hypertensive women are common attenders in GP. They are at increased risk for developing diabetes and cardiovascular disease (CVD), unless efficient preventive actions are organised [27]. The problem is that the available score systems for CV risk assessment are not sensitive enough to ensure accurate risk stratification of this population group [28]. Thus, research with the potential to provide general practitioners with tools for fast recognition of middle-aged women at high CV risk, would make a substantial contribution to CVD prevention, because in women, as it is in men, CVD are the main cause of death [27].

Hypertension is the main CV risk factors, for its high prevalence in population and great impact on CV morbidity and mortality [29]. There are close, although insufficiently understood relationships between increased body weight (general obesity), abdominal (central) obesity and hypertension [30]. Hypertension is one of the most prominent components of the metabolic syndrome (MS) [31]. It is defined as a cluster of CV risk factors that includes abdominal obesity (indicated with increased waist circumference), glucose intolerance or diabetes type 2 and dyslipidemia characterised with increased triglycerides and decreased HDL-cholesterol. MS, superimposed to hypertension, significantly amplifies CV risk [32].

There are many concerns associated with characteristics of hypertensive women in the age around menopause. In early postmenopausal women, hypertension was found to more oftenly present as a part of MS than as an isolated disease [33]. Transition from pre- to postmenopause, around the age of 50, is the critical period in women’s life, when obesity, hypertension and other CV risk factors start to emerge. Also prevention of CVD is then most useful [34, 35]. However, there is a large amount of variation in CV risk factors expression, because of intensive emotional and lifestyle changes taking place during this transition and of possible discordance between the chronological age and the reproductive age at the time of menopause, that may also influence these variations [36, 37]. Several medical conditions and biohumoral alterations, apart from CVD, including e.g. chronic low grade inflammation, renal function decline, anxiety/depression, sleep and cognitive disorders, have been identified to coexist with MS, contributing to variations in phenotypes and CV risk profiles of patients with MS [38–42].

Components of MS and the role they have in development of CVD were found to be gender dependent, indicating the need for different criteria of MS for men and women [43]. There are several working definitions of MS that differ to each other to some extent, both in composition of the components of MS and in their cut-off values [44]. These definitions are clinical constructs, built upon the cut-off for increased CV risk in the referent populations. There is a long lasting debate on whether MS is a syndrome or a mixture of low related phenotypes, the composition of which can vary in different population groups [45].

### Objectives

We setted up two main objectives. The first one was to evaluate the appropriateness of the standard definition of MS to apply to this specific, narrowly defined population group. Results are expected to improve our understanding on relationships between hypertension and other components of MS and other CV risk factors, in menopausal women. This knowledge might be useful in improving decision making on this complex issue. The second objective was to improve recognition of women at high CV risk, by identifying relevant markers and phenotypes, including also comorbidities and broader social context, in addition to components of MS. In particular, we wanted to assess the feasibility of data available in GP eHRs, completed with patients interview, to support this phenotype profilling process. Results are expected to inform the composition of the standard data record in GP eHRs and future research. Finally, through this analysis, we wanted to explore the potential of using a combination of methods in getting useful information from the available data.

## Methods

### Study population and the sample

**Fig. 1:**
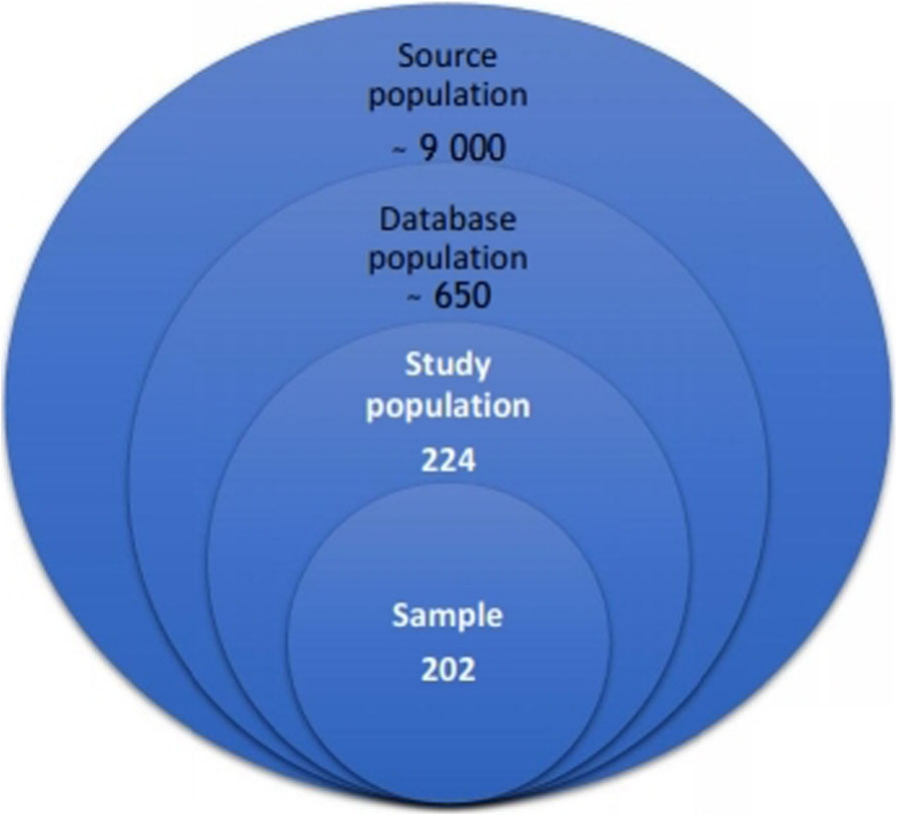
Study population and the sample

The study was performed in a GP setting, in an urban-rural area (12.000 inhabitants), eastern Croatia, Central European region. Data were used from six practices located in the same health centre (source population: roughly 9.000 adult patients) (Fig. 1). As evidence says, general practitioners who work in the close vicinity use similar professional vocabulary and content meaning of encoded terminology, that can contribute to data consistency [46]. In addition, physicians who participated in this study were all specialists in GP, with more than 15 years of work experience, that means, skilled in diagnosis and evidence-based prescribing, that could also contribute to the accuracy of data recording.

**Fig. 2:**
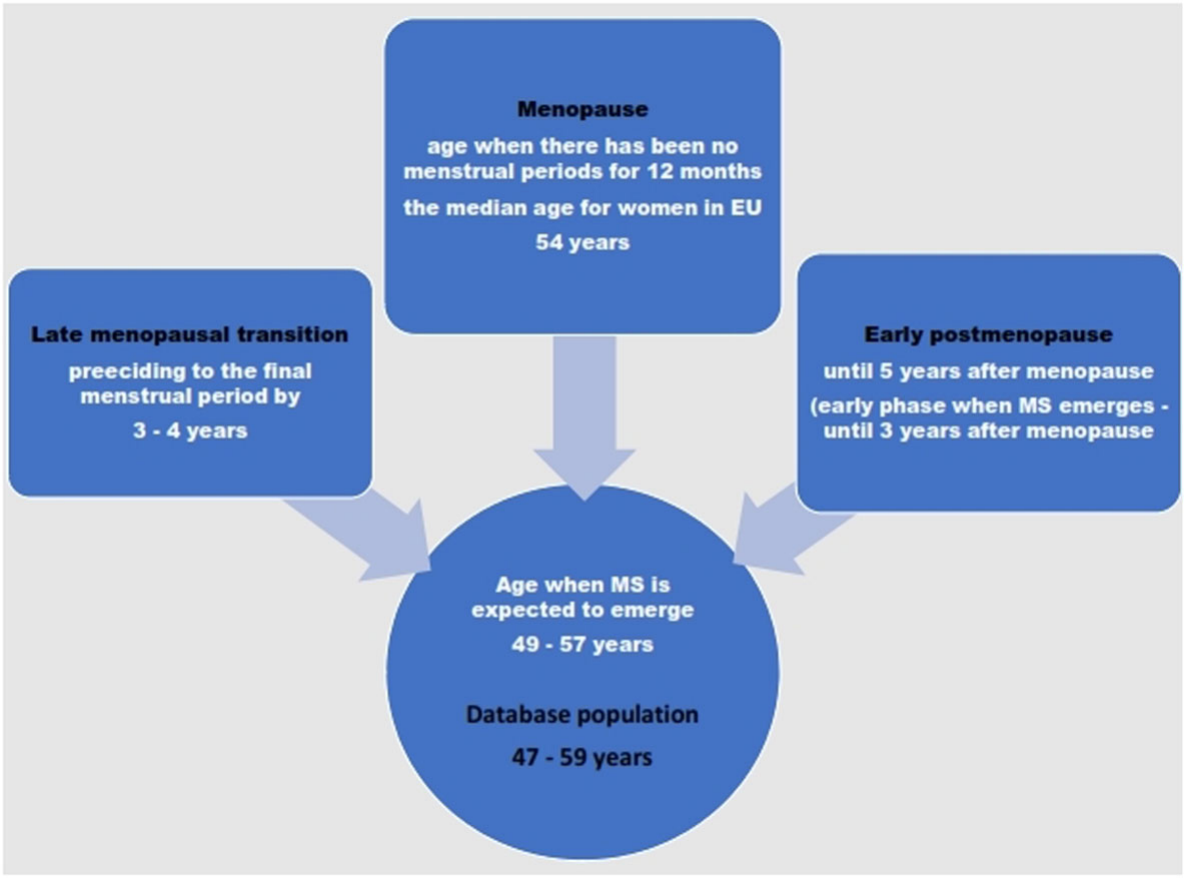
Evidence that guided the choice of criteria for the database population

As the database population, we used women old 47–59 years (650 subjects included) (Fig. 1). We chose this age range as the population selection criterium, being guided with the knowledge on the chronological age that, in women in EU countries, best matches the reproductive periods when MS is most likely to emerge (Fig. 2) [47–49].

As the study population, we used only those women from this age range who were diagnosed with hypertension (*N* = 224) (Fig. 1). Five of them reported surgically induced menopause and one reported the use of the hormone replacement therapy. They were excluded from the study. Fourteen women did not respond to our call for interview. For two cases, data were incomplete. Thus, the final number of women, included in the study, was 202 (Fig. 1).

### Study design

A retrospective and cross-sectional, observational and exploratory study, prepared according to the guidelines for using observational routinely-collected health data (RECORD statement) [50]. On the input data set, composed mostly of data from GP eHRs, we applied a combination of statistical and data mining methods that we supposed appropriate for the objectives. A minor part of data, for which evidence showed their association with MS but that have not been systematically recorded in GP eHRs, we obtained by patients’ interview. We also included anthropometric measurements as a part of the interview. Candidate women, we invited by phone, or by using the mobile short message service (sms), or we asked them for the interview when they came to the regular encounter. The team leader physicians had been previously instructed for conducting the interviews.

### Croatian PHC ICT system and GP eHRs

**Fig. 3:**
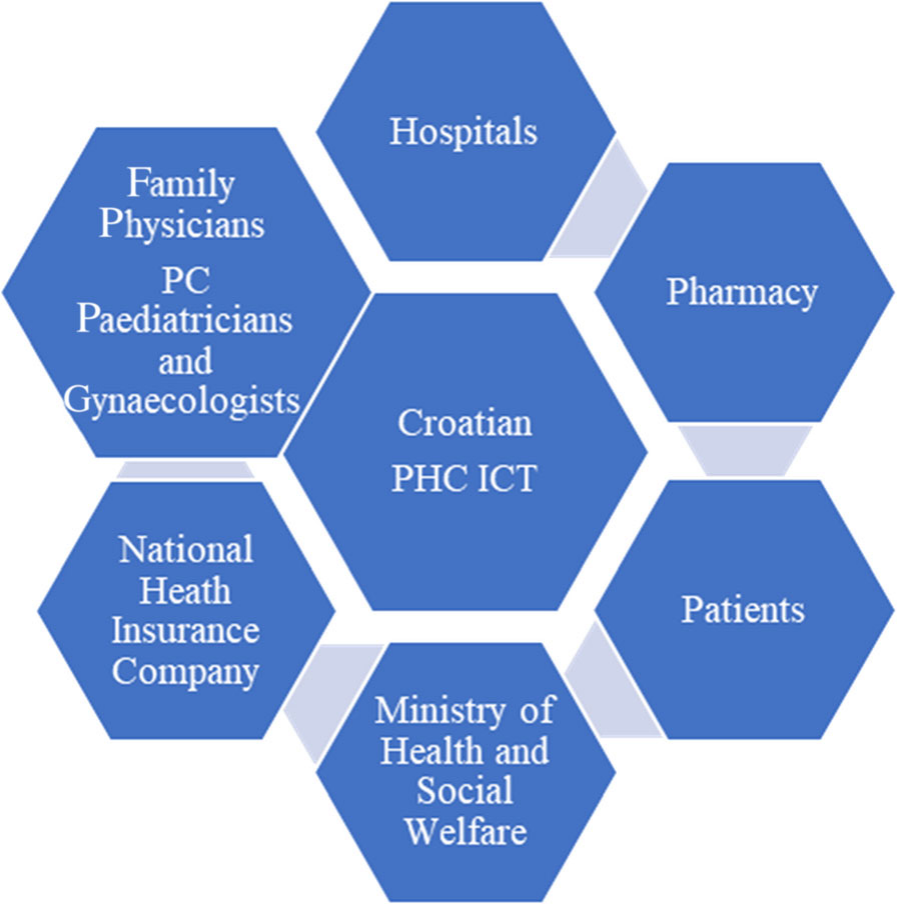
The Croatian Primary Health Care (PHC) Information Communication Technology (ICT) System

In Croatia, PC services have the gatekeeping role. The ICT system and eHRs were firstly developed in PHC and GP settings and boosted several times, primarily to improve connections within the PC services (Fig. 3) [51]. Recently, also e-referrals to specialists have been introduced. In order to improve the quality of care, the panel support tools for chronic disease surveillance and preventive check ups, have been established.

The ICD-10 code (International Statistical Classification of Diseases and Related Health Problems, 10th Revision) is used to support patient encounters. In order to support the prescription procedure, the medication list, together with the prescription rules, are available online to each PC physicians and regulary updated. Reference ranges of blood tests are incorporated in the primary laboratory test report templates.

The main barriers for using data from GP eHRs for research, in Croatia, include a large number of working applications, the lack of eHR data standards and the lack of networking into the common research platform.

### Data set description

The input data set was composed of a total number of 62 parameters, of which 50 parameters were used from GP eHRs (Table 1) and 12 were obtained by patients’ interview (Table 2).

**Table 1:** Parameters used from GP eHRs and their abbreviations and descriptions

	Parameter	Description
Age	Age (years)	Numeric < 47, 59>
Hypdu	Hypertension duration (years)	Nominal {< 5, 5–10, > 10}
Hypre	Hypertension regulation	Binary {Normal /No}
DGDM	Dg Diabetes mellitus	Binary {Yes/No}
DMdu	Diabetes duration (years)	Nominal {0, 1–4, 5–10, > 10}
DMtr	Diabetes treatment (oral antidiabetics, oral antidiabetics+insulin, insulin)	Nominal {no, or, or/and, and}
DMco	Diabetes complications: macrovascular (coronary heart disease, cerebrovascular disease, peripheral arterial disease) and microvascular (retinopathy, proteinuric nephropathy, low extremity ulcers)	Nominal {no, mac, mic}
chre	Chronic respiratory diseases (asthma, COPB)	Binary {Yes/No}
allre	Respiratory allergic diseases (rhinitis)	Binary {Yes/No}
CHD	Chronic heart disease (heart failure, cardiomyopathy)	Binary {Yes/No}
CoHD	Coronary heart disease (angina, infarct myocardii, revascularization)	Binary {Yes/No}
CV	Cerebrovascular disease (transitory ischaemic attack, cerebrovascular insult, carotid atherosclerosis)	Binary {Yes/No}
PAS	Peripheral artery disease	Binary {Yes/No}
GI	Upper gastro-intestinal disease (reflux, gastritis, duodenal ulcer)	Binary {Yes/No}
infbo	Inflammatory bowel disease (Crohn disease, ulcerative collitis)	Binary {Yes/No}
hep	Chronic liver disease (chronic virus hepatitis, cirrhosis)	Binary {Yes/No}
mal	Malignant disease (not patients at terminal stage and under therapy)	Binary {Yes/No}
op	osteoporosis	Binary {Yes/No}
OA	Osteo-arthritis	Binary {Yes/No}
Sis	Collagenosis (rheumatoid arthritis, spondylarthritis, systemic lupus, systemic sclerosis, MCTD, dermatomyositis)	Binary {Yes/No}
Uro	Chronic urogenital disease (chr cystitis, pyelonephritis)	Binary {Yes/No}
cogn	Cognitive impairment &dementia	Binary {Yes/No}
thy	Endocrine disease (the thyroid gland disease)	Binary {Yes/No}
depr	Anxiety&depression	Binary {Yes/No}
psy	Psychosis	Binary {Yes/No}
derm	Chronic skin disease (psoriasis, chronic ezcema)	Binary {Yes/No}
chdi	A total number of chronic diseases	Numeric < 1, 10>
sta	statins ≥3 months)	Binary {Yes/No}
BB	beta-blockers (≥ 3 months)	Binary {Yes/No}
anco	anticoagulant drugs (≥ 3 months)	Binary {Yes/No}
anal	Analgesics/Non-steroidal anti-inflammatory drugs (frequent use: ≥3 months in the past year)	Binary {Yes/No}
anbi	Antibiotics (≥2 times per year)	Binary {Yes/No}
met	Metformin (≥ 3 months)	Binary {Yes/No}
Ace	ACE-INH (or AT-receptor antagonist) ≥ 3 months	Binary {Yes/No}
Drug	A total number of oral drugs	Numeric < 1, 9>
FGlu	Fasting glucose (mmol/L)	Numeric < 4.00, 13.00>
TG	Triglycerides (mmol/L)	Numeric < 0.42, 4.49>
cho	Total cholesterol (mmol/L)	Numeric < 3.50, 8.20>
LDL	LDL-cholesterol (mmol/L)	Numeric < 1.02, 5.79>
HDL	HDL-cholesterol (mmol/L)	Numeric < 0.80, 2.60>
cre	Serum creatinine (μmol/l)	Numeric < 43.00, 110.00>
GFR	Glomerular filtration rate (mL/min/1.73 m^2^) (MDRD^a^ on-line calculator)	Numeric < 40.9, 143.3>
Le	Leukocytes number (10^9^/L)	Numeric < 3.90, 11.50>
CRP	C-reactive protein (mg/L)	Numeric < 0.20, 11.50>
Mo	Monocytes% (in blood differential count)	Numeric < 0.00, 7.00>
Ly	Lymphocytes % (in blood differential count)	Numeric < 5.00, 48.00>
Er	Erythrocytes number (10^12^/L)	Numeric < 3.66, 5.80>
Hb	Haemoglobin (g/L)	Numeric < 106.00, 155.00>
Htc	Haematocrit (%)	Numeric < 0.29, 0.50>
Fe	Serum iron (μmol/l)	Numeric < 6.30, 26.80>

^a^Modification of Diet in Renal Disease - a formula used for calculation of glomerular filtration rate

**Table 2:** Parameters obtained by patients interview and their abbreviations and descriptions

	Parameter	Description
wai	Waist circumference (cm)	Numeric < 78, 120>
BMI	BMI (height, weight) (kg/m^2^)	Numeric < 21.11, 38.28>
smo	Cigarette smoking (Yes, stopped, No)	Nominal {Yes, sto, No}
alc	Usual in house alcohol use (for two or more days a week)	Binary {Yes/No}
fhis	Family history of CVD: Yes/No	Binary {Yes/No}
soc	Socio-economic status (good, intermediate, low)	Nominal {g, in, lo}
meno	Menopause duration (age of onset/years after menopause)	Nominal {no, < 1, 1–3, > 3}
chil	Number of children born	Numeric < 1, 5>
abor	Number of abortuses	Numeric < 0, 2>
sle	Sleep quality (good/bad)	Binary {g, b}
phy	Physical activity level (good, intermediate/low)	Nominal {g, in/lo}
HRT	Hormonal replacement therapy: Yes/No	Not applicable (1 case)

From GP eHRs, only structured data were used, including: 1) demographics, 2) diagnoses of chronic diseases, 3) names of medications in a continuous use and 4) results of laboratory tests (Table 1). The high level of data completeness (only two cases of the study populations had incomplete data) was assured according to the fact that this data type are being systematically recorded.

To diagnose some well-defined chronic medical conditions, but for which the diagnosis coding system does not proved the suitable framework, such as stages of chronic renal impairment, impaired glucose tolerance and dyslipidemias, criteria for cut-off values were used from the current international guidelines (Table 3) [28, 52–54].

**Table 3:** Definitions and grading of some medical conditions

Parameters	Definitions or grading
Stages of chronic renal impairment	Grading is based on using GFR^a^ cut-off values - a measure of renal function decline that is derived from data indicating sex, age, weight and serum creatinine (according to the online available MDRD^b^ formula)
Impaired fasting blood glucose Diabetes type 2 diagnosis	Fasting blood glucose ≥6.1 < 7.0 mmol/L Fasting blood glucose ≥7.0 mmol/L
Normal blood lipids values	Total cholesterol < 5.0 mmol/L LDL-cholesterol ≤3.0 mmol/L HDL-cholesterol ≥1.2 mmol/L (F) Triglycerides ≤1.7 mmol/L
Categories of BMI	BMI < 19 - malnutrition BMI 19–24.9 - normal weight BMI 25–29.9 - overweight BMI ≥30 - obesity
Physical activity level	Good: sport activities, farming or out of house work (at least twice a week) Intermediate: housekeeping, walking for an hour or more (at least twice a week) Low: sedentary lifestyle and lower-energy in house activities
Socio-economic status	Good: bigger house or flat residency and income at the average or above the average for Croatia Intermediate: smaller house or flat residency and income below the average for Croatia but still sufficient to meet the existential needs Low: ^c^poor housing and low income, below the existential needs
Positive family history on CVD	Defined as an early onset of coronary heart disease, at or before 50 years of age, for the father and brothers, and at or before 60 years of age, for the mother and systers

^a^Glomerular filtration rate

^b^Modification of Diet in Renal Disease

^c^Only two subjects registered

To improve patients phenotype profiling, we added also medications to the input data set. We used information only on those medications that are known to have the effects on the development of MS or CVD, including: statins (cholesterol lowering drugs), beta-blockers, ace-inhibitors/receptor blockers, anticoagulants, analgesics or non-steroidal anti-inflammatory drugs (NSAD), antibiotics and metformin (first choice oral antidiabetic drug) [55–57].

We included laboratory tests in the input dataset, to identify possible haematology and biochemical disorders that in hypertensive menopausal women determine MS. Of laboratory findings, we used those ones that were old no more than a year and that were performed as a part of the periodic chronic disease surveillance or preventive check ups.

By patients’ interview, information were gain on factors known to influence MS, but for which records in GP eHRs were either incomplete or missing (Table 2) [58–60]. Definitions and grading for some of these factors are provided in the Table 3. To diagnose the positive family history on CVD, definition was used from the guidelines [28]. To identify the physical activity level, the scale was used from papers published on frailty syndrome, but modified, to fit the habits of the local elderly population [61]. Description of the socio-economic status relied on the authors’ subjective assessment of the living conditions of elderly people in the local community. Self-reported information on impaired sleep patterns, in the last month, was used to diagnose sleep disturbance. Anthropometric measurements, waist circumference and weight and height (for calculation of BMI), were taken from participants during the interview. The WHO classification of categories of BMI, cited elsewhere, was used to differentiate between women with normal weight and those being overweight or obese.

To diagnose MS, we used the definition of the International Diabetes Federation (IDF) (2005), because it fitted well to the objective, to identify women at high risk for CVD [62]. Namely, this definition is sensitive on the abdominal type of obesity and considers also diabetics with MS. In addition, it relies on data available in GP eHRs.

The IDF definition of the metabolic syndrome - the female gender option.

Waist circumference ≥ 80 cm + 2 out of 4 criteria:

1. Diagnosis of hypertension
2. Triglycerides > 1.70 mmol/L
3. HDL-cholesterol < 1.3 mmol/L
4. Fasting glucose ≥5.6 mmol/L or the diagnosis of diabetes

### Methods for data analysis

#### Basic statistics. Differences in distributions

The Shapiro-Wilks normality test was used to determine whether or not numerical parameters take the normal distribution [63]. For normally distributed numerical parameters, the parametric 2-sample Welch’s t-test was used to analyse differences in distributions between women with and without MS, otherwise it was the non-parametric Mann-Whitney-Wilcoxon test [64]. Distributions of categorical parameters were assessed by using the Pearson’s chi-squared test, except when the expected number of observation was less than 5, when the Fisher’s Exact test was more appropriate. For all tests, the level of significance was set up at 0.05.

#### Estimation of cut-off values

The Youden method, based on calculation of the Youden’s index, *YI*(*c*) = *max*_*c*_(*Se*(*c*) + *Sp*(*c*) − 1), was used to identify cut-off values of numerical parameters that were showed significant in the analysis of differences [65]. Statistical measures: sensitivity, specificity, positive predictive value (PPV) and negative predictive value (NPV) were used to measure the prediction accuracy for MS of the estimated cut-off values. This method was necessary for the assessment of the appropriateness of criteria provided by the conventional definition of MS to comply with characterististics of MS in the group of hypertensive menopausal women.

#### Graphical methods for data presentation

Some important numerical parameters, of those found significant in analysis of differences, and their cut-off values, were presented also graphically, as box plot graphs.

Bar graphs were used to make visible frequency distributions of women with and without MS according to the time-dependent categories of the parameters indicating: menopause, hypertension and diabetes duration. These bar graphs added value to information obtained by the LR model, on the effect of these parameters on MS.

#### Multiple logistic regression

Four models of multiple logistic regression (LR) were developed to determine relationships between particular groups of parameters, indicating different aspects of the patient phenotypes, and the presence of MS, in hypertensive menopausal women. The 95% confidence interval (CI) was used to estimate the precision of odds ratio (OR). The McFadden’s R squared test was used to measure the predictive power of the LR models [66].

Four LR models were defined as:

1. metabolic components of MS and associated biohumoral disorders presented as haematological and biochemical tests (parameters: BMI, wei, Fglu, TG, HDL, cho, LDL, cre, GFR, CRP, Le, Mo, Ly, Htc, Er, Hb, Fe)
2. comorbidities, medical histories, socio-economic and lifestyle factors (parameters: CHD, CoHD, infbo, cogn, depr, sle, chdi, drug, OA, op, thy, fhis, chil, abor, soc., phy, smo, alc)
3. medications (parameters: sta, BB, met, anal, ace, anbi, anco)
4. age, menopause duration, hypertension duration and regulation, diabetes diagnosis, diabetes duration, treatment and complications (parameters: age, meno, Hypdu, Hypre, DGDM, DMdu, DMco, DMtr)

#### Decision trees method

The C5.0 algorithm, an advanced binary decision trees (DT) method, was used to define simple, practically useful rules, to help general practitioners recognise hypertensive menopausal women with MS [67]. Characteristics of this method, such as a small number of rules that it produce, made it appropriate for the development of rules that draw upon the full-range of data used in the input.

In order to improve the diagnostic capacity of these rules, to go beyond the framework of the conventional definition of MS, two DT models have been performed: 1) on the full-range of data and 2) on the input data set after the parameters indicating conventional components of MS, including: waist circumference, BMI, triglycerides, HDL-cholesterol and fasting blood glucose, had been removed.

## Results

### Differences in distributions

Women with MS, compared to those without, showed significant differences in a wide range of numerical (Table 4, bolded) and categorical parameters (Table 5, bolded).

**Table 4:** Differences in distributions of numerical parameters between hypertensive menopausal women with and without metabolic syndrome

Parameter	Mean (±Standard deviation)		Variance		*p*-value**
	MS (yes)	MS (no)	MS (yes)	MS (no)	
BMI (kg/m^2^)	29.02 ± 3.670	24.76 ± 2.606	13.467	6.790	**5.27e-16**
TG (mmol/L)	1.939 ± 0.581	1.434 ± 0.304	0.338	0.092	**1.27e-14**
wai (cm)	93.48 ± 8.639	84.74 ± 5.280	74.636	27.872	**5.84e-14**
cho (mmol/L)	6.315 ± 0.885	5.25 ± 0.735	0.783	0.541	**1.52e-13**
LDL (mmol/L)	3.366 ± 1.023	2.552 ± 0.824	1.046	0.680	**3.82e-09**
FGlu (mmol/L)	5.924 ± 1.031	5.333 ± 0.402	1.063	0.161	**7.35e-08**
chdi	5.214 ± 1.445	4.304 ± 1.458	2.092	2.127	**4.07e-05**
CRP (mg/L)	3.295 ± 1.575	2.57 ± 1.275	2.481	1.625	**0.0018**
Drug	4.321 ± 1.618	3.565 ± 1.728	2.619	2.985	**0.0021**
Age	52.79 ± 1.977	52.07 ± 2.024	3.908	4.098	**0.0054**
cre (μmol/l)*	79.75 ± 11.545	75.81 ± 12.534	133.282	157.096	**0.0319**
Er (10^12^/L)	4.641 ± 0.361	4.521 ± 0.395	0.130	0.156	**0.0292**
Htc (%)	0.3899 ± 0.043	0.3733 ± 0.033	0.002	0.001	**0.0302**
Hb (g/L)	136 ± 9.127	133.7 ± 8.375	83.307	70.137	0.0659
GFR (mL/min/1.73 m^2^)	71.44 ± 13.102	74.71 ± 15.537	171.657	241.402	0.0816
Fe (μmol/l)	16.16 ± 3.338	15.38 ± 2.614	11.143	6.832	0.1684
Ly (% in blood differential count)	22.61 ± 7.928	20.54 ± 4.337	62.855	18.811	0.2123
Chil	2.023 ± 0.956	1.884 ± 0.932	0.915	0.869	0.2869
Abor	0.1832 ± 0.443	0.2174 ± 0.481	0.197	0.231	0.6139
Le (10^9^/L)	6.676 ± 1.488	6.777 ± 1.349	2.213	1.820	0.9261
HDL (mmol/L)	1.346 ± 0.424	1.306 ± 0.329	0.179	0.108	0.9263
Mo (% in blood differential count)	3.023 ± 1.551	3.043 ± 1.288	2.407	1.660	0.9584

*Welch two sample t-test; ** significant parameters were marked with bolded fonts

**Table 5:** Differences in distributions of categorical parameters between hypertensive menopausal women with and without metabolic syndrome

Parameter	*p*-value**	Parameter	*p*-value	Parameter	*p*-value	Parameter	*p*-value
sta	**3.468e-14**	sle	**0.005866**	soc	0.2193	anco	0.6943*
DMtr	2.452e-10*	CoHD	**0.005915**	thy	0.2198	Uro	0.8019
DMdu	2.109e-09*	Hypdu	**0.008909**	smo	0.2241	GI	0.9406
DGDM	**4.043e-07**	Hypre	**0.01481**	mal	0.2317	allre	0.9653
met	**3.789e-07**	DMco	0.0211*	anbi	0.3154	CV	1*
BB	**1.542e-05**	infbo	**0.03989**	OA	0.4277	Sis	1*
depr	**0.0003668**	fhis	0.07321	op	0.4543	ace	1*
meno	0.001047*	psy	0.07358*	hep	0.4987	phy	0.9952
CHD	0.004887*	anal	0.09414	PAS	0.5352	chre	0.6319
cogn	**0.005871**	alc	0.09775	derm	0.207*		

*Fisher exact test; **significant parameters were marked with bolded fonts

### Estimation of cut-off values

Table 6 represents cut-off values of those numerical parameters that in the Table 4 have been presented as significant. Parameters: indicating BMI, waist circumference, total serum cholesterol and triglycerides, showed best statistical performance measures of their cut-off values (bolded).

**Table 6:** Cut-off values of numerical parameters found significant in the analysis of differences

Parameter	Cut-off	Sensitivity	Specificity	Positive predictive value	Negative predictive value	False positive results	False negative results
Age	55	0.290	0.899	0.844	0.400	7	93
chdi	5	0.702	0.580	0.760	0.506	29	39
Drug	3	0.870	0.348	0.717	0.585	45	17
cre (μmol/l)	77	0.595	0.623	0.750	0.448	26	53
CRP (mg/L)	2.3	0.733	0.565	0.762	0.527	30	35
Er (10^12^/L)	4.51	0.656	0.551	0.735	0.458	31	45
BMI (kg/m^2^)	25.51	**0.855** ^**a**^	**0.797**	**0.889**	**0.743**	**14**	**19**
cho (mmol/L)	6	**0.740**	**0.754**	**0.851**	**0.605**	**17**	**34**
wai (cm)	89	**0.740**	**0.797**	**0.874**	**0.618**	**14**	**34**
TG (mmol/L)	1.7	**0.718**	**0.942**	**0.959**	**0.637**	**4**	**37**
LDL (mmol/L)	3.1	0.573	0.870	0.893	0.517	9	56
FGlu (mmol/L)	5.7	0.542	0.884	0.899	0.504	8	60
Htc (%)	0.41	0.359	0.855	0.825	0.413	10	84

^a^Bolded parameters show the best cut-off values with respect to statistical performance measures

### Graphical presentations of some results

**Fig. 4:**
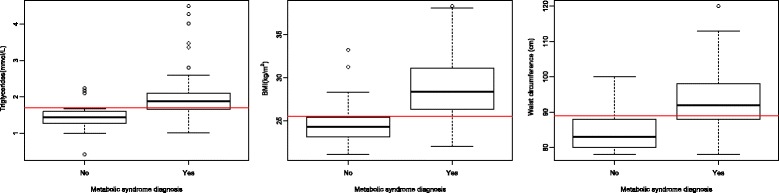
Graphical presentations of differences in distributions of numerical parameters: triglycerides (left), BMI (middle) and waist circumference (right) with respect to the presence or not of the diagnosis of metabolic syndrome

How well cut-off values of the significant numerical parameters: triglycerides, BMI and waist circumference, discriminate between hypertensive menopausal women with and without MS, it is better visible when differences in these parameters are presented graphically, as box plot graphs (Fig. 4, left, middle, right).

**Fig. 5:**
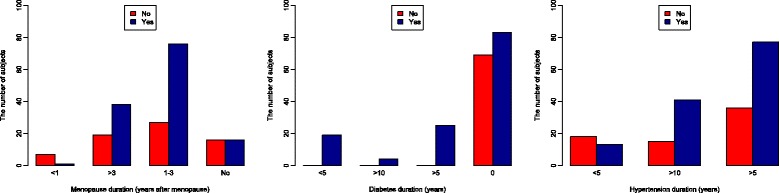
Graphical presentations of frequency distributions of women with and without MS according to the categories of parameters: menopause duration (left), diabetes duration (middle) and hypertension duration (right)

Frequency distributions of women with and without MS according to the categories of parameters: menopause duration, diabetes duration and hypertension duration, were presented graphically, as bar graphs (Fig. 5, left, middle, right).

### LR models

The overall predictive accuracy of this LR model is 70.1%. Parameters significantly associated with MS indicate: BMI, fasting blood glucose, triglycerides, total serum cholesterol, leukocytes number and monocytes % in blood differential count. The parameter indicating haematocrit, although showed no significant association with MS, is presented with the big OR (Table 7).

**Table 7:** Logistic regression model with included parameters indicating conventional components of metabolic syndrome and associated haematology and biochemical disorders

Parameter				CI	
	z value	Pr(>|z|)	OR	5%	95%
BMI	4.24	0.220e-04***	2.661	1.821	3.889
wai	−0.39	0.694	0.970	0.856	1.099
FGlu	2.45	0.014*	5.621	1.762	17.928
TG	2.49	0.012*	18.455	2.689	126.658
HDL	−1.04	0.299	0.4268	0.110	1.643
cho	3.29	0.001**	10.897	3.295	36.031
LDL	−0.32	0.749	0.874	0.437	1.746
cre	−0.41	0.684	0.967	0.846	1.105
CRP	0.53	0.594	1.181	0.705	1.981
Ly	1.61	0.107	1.116	0.997	1.249
Er	1.25	0.212	5.788	0.569	58.821
Htc	1.04	0.298	**6.274e04**	0.159e-02	2.473e12
GFR	−1.57	0.116	0.893	0.793	1.005
Le	−2.44	0.014*	0.444	0.256	0.767
Mo	2.19	0.028*	1.871	1.168	2.998
Hb	−1.38	0.166	0.932	0.858	1.013
Fe	0.02	0.982	1.003	0.784	1.283

Bolded: parameters with the levels of significance: *** 0.001 ** 0.01 * 0.05 or big ORs

The overall predictive accuracy of this LR model is 29.9%. Parameters that showed significant associations with MS or the big ORs indicate: diagnosis of anxiety/depression, alcohol use, intermediate to low socio-economic status, diagnoses of CVD (including both chronic heart disease and coronary heart disease), diagnosis of inflammatory bowl disease and psychotic disease (Table 8).

**Table 8:** Logisitc regression model with included parameters indicating comorbidities, medical histories, socio-economic and lifestyle factors

Parameter				CI	
	z value	Pr(>|z|)	OR	5%	95%
CHD Yes	0.01	0.990	**2.147e07**	0.000	Inf
CoHD Yes	0.01	0.989	**3.103e07**	0.000	Inf
infbo Yes	0.00	0.996	**1.096e-08**	0.000	Inf
cogn Yes	1.35	0.177	3.218	0.772	13.404
depr Yes	2.84	0.0045**	6.572	2.207	19.573
psy Yes	0.00	0.997	**1.217e07**	0.000	Inf
sle g	0.45	0.652	1.200	0.615	2.343
chdi	1.62	0.105	1.355	0.995	1.847
Drug	1.96	0.050	1.262	1.037	1.536
OA Yes	0.41	0.679	1.195	0.587	2.430
op Yes	−0.80	0.422	0.580	0.190	1.769
thy Yes	0.92	0..357	1.710	0.655	4.464
phy g	0.43	0.666	0.471	0.203	1.666
smo sto	−0.73	0.464	0.672	0.275	1.640
smo Yes	−1.73	0.083	0.466	0.226	0.962
alc Yes	2.67	0.007**	16.170	2.903	90.047
soc in	2.29	0.022*	3.956	1.472	10.631
soc lo	0.01	0.995	**5.177e08**	0.000	Inf
fhis Yes	0.53	0.595	1.265	0.610	2.622
chil	1.42	0.155	1.349	0.954	1.910
abor	−0.72	0.473	0.738	0.367	1.480

Bolded: parameters with the levels of significance: ** 0.01 * 0.05 or big ORs

The overall predictive accuracy of this LR model is 40.9%. All parameters from the input indicating medications were selected in the model, but with variable contributions (ORs) to the diagnosis of MS. Parameters that showed significant associations with MS indicate: use of statins, metformin and beta-blockers (Table 9).

**Table 9:** Logistic regression model with included parameters indicating medications

Parameter				CI	
	z value	Pr(>|z|)	OR	5%	95%
Sta Yes	5.90	0.36e-08***	15.829	7.329	34.186
BB Yes	2.04	0.040*	2.685	1.213	5.945
met Yes	3.54	0.4e-03***	44.471	7.622	259.450
anal Yes	1.01	0.311	1.599	0.745	3.433
ace Yes	−1.62	0.106	0.375	0.138	1.017
anbi Yes	−1.70	0.088	0.426	0.187	0.971
anco Yes	− 184	0.066	0.126	0.019	0.805

Bolded: parameters with the levels of significance: *** 0.001 * 0.05

The overall predictive accuracy of this LR model is 27.9%. The parameter that showed significant association with MS indicates menopause of 1–3 years of duration. Parameters that showed no significant associations with MS but that have the big ORs, indicate diagnosis of diabetes and diabetes duration of less than a year (Table 10).

**Table 10:** Logistic regression model with included parameters indicating: age, menopause duration, hypertension duration and regulation, diabetes diagnosis, duration, treatment and complications

Parameter				CI	
	z value	Pr(>|z|)	OR	5%	95%
Age	0.60	0.548	1.066	0.894	1.242
Meno > 3	1.73	0.084	6.989	1.098	44.453
Meno 1–3	2.18	0.029*	11.192	1.810	69.181
Meno No	1.28	0.200	4.522	0.652	31.341
Hypdu > 10	1.05	0.296	1.804	0.712	4.570
Hypdu > 5	0.75	0.453	1.438	0.648	3.195
Hypre Normal	−1.90	0.057	0.320	0.119	0.858
DGDM Yes	0.00	0.997	**0.646e-08**	0.000	In0
DMdu > 10	0.00	1.000	1.477	0.000	Inf
DMdu > 5	0.00	1.000	1.094	0.000	Inf
DMdu 0	0.00	0.996	**0.971e-16**	0.000	Inf
DMco mic	0.00	1.000	1.321	0.000	Inf
DMco No	0.00	1.000	1.088	0.000	Inf
DMtr or	0.00	1.000	0.938	0.000	Inf
DMtr or/i	0.00	1.000	2.103	0.000	Inf

Bolded: parameters with the levels of significance: * 0.05 or big ORs

### DT models

**Fig. 6:**
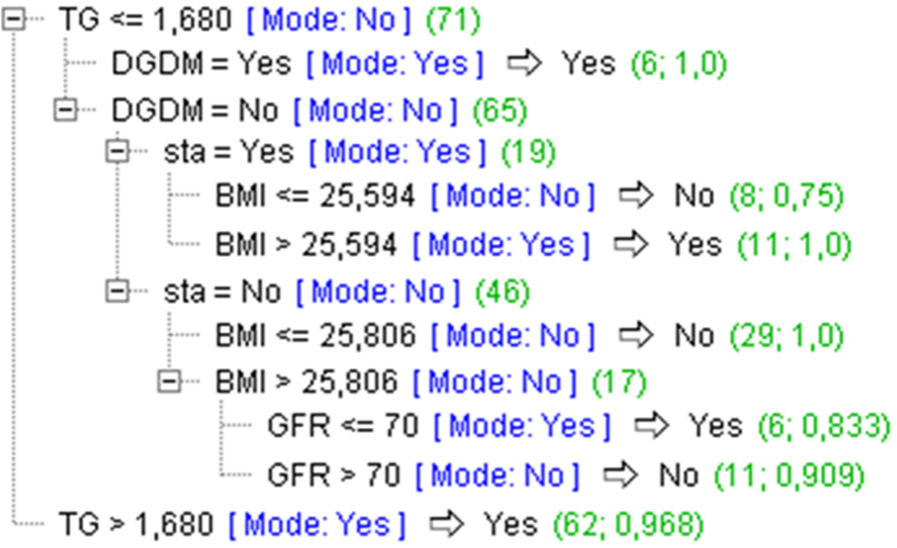
Decision trees model with all parameters included

The overall predictive accuracy of this model is 91.04%. Two major group of rules (phenotypes) were identified: 1) when triglycerides are increased (TG > 1.68) (confirms the diagnosis of MS with the accuracy of prediction of 96.8%) and 2) a set of rules when triglycerides are not increased (TG ≤ 1.68) (Fig. 6).

When triglycerides are not increased, phenotypes that can be used to identify hypertensive menopausal women with MS, include: diagnosis of diabetes (*N* = 6, accuracy 100%); otherwise, increased BMI (> 25.59) and statins use (*N* = 11, accuracy 100%) or increased BMI (> 25.80) and mild renal impairment (GFR ≤ 70) (N = 6, accuracy 83.3%).

**Fig. 7:**
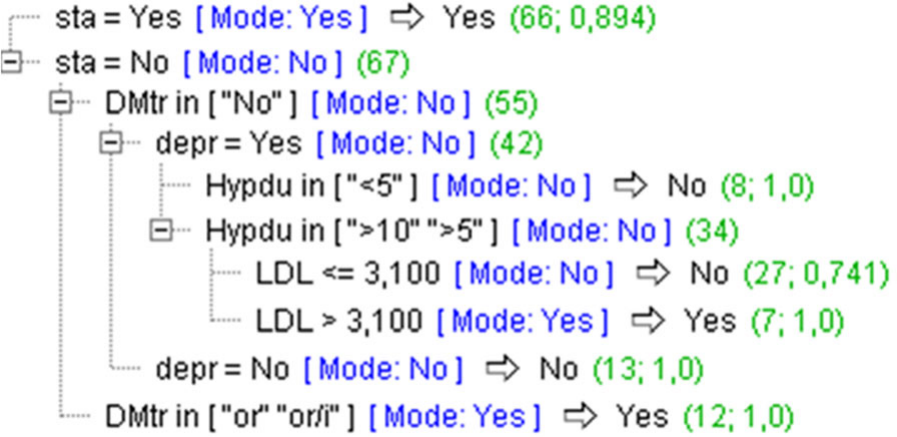
Decision trees model with excluded parameters closely related to the conventional definition of metabolic syndrome: waist circumference, BMI, triglycerides, HDL-cholesterol and fasting glucose

The overall predictive accuracy of this model is 89.55%. Two major group of rules (phenotypes) were identified, based on information of whether or not women use statins (Fig. 7).

By the single statement, on statins use, it is possible to recognise a half of the total number of women with MS (66 out of 133), with the accuracy of recognition of 89.4%.

Women with MS who do not take statins, can be recognised according to the phenotypes: 1) treated diabetes, corresponding with overt diabetes (*N* = 12, accuracy 100%) or 2) not treated diabetes, corresponding with a new onset diabetes, to coexist with anxiety/depression, hypertension of more than 5 years of a duration and increased LDL-cholesterol (> 3.1 mmol/L) (*N* = 7, accuracy 100%).

## Discussion

### General characteristics of the study population

Chronological age of women in the sample when MS is most likely to emerge was found to be 50–55 years (exactly 50.8–54.8), with the average age of 52–53 years (Table 4). This age range can be used as the screening criterium for women with MS and, in general, for those who are at high CV risk. This is supported with the result of a high percentage (65.8% or 133/202) of women with MS, that is higher than the large-scale studies showed for the general population and even higher than it was found for the selected population of hypertensive patients with uncontrolled blood pressure [68, 69]. In addition, a high percentage of these women with MS also had diabetes (27.2% or 55/202). This percentage is higher than it has been reported e.g. for diabetics in older Croatian population [70]. Taken together, these results implicate the high-grade CV risk of women in the sample. These results are even more remarkable, when taking into account that almost all diabetics had MS (53/55), according to the evidence that diabetics with superimposed MS yield more CV risks [71].

### Anthropometric measures and other conventional components of MS

Waist circumference, a measure of the central (abdominal) obesity, is a part of the most of available definitions of MS [44]. On the contrary, a measure of the general obesity, indicated with BMI > 30, makes part of only one definition, in line with evidence that increased body weight may not always be associated with MS [68]. Starting from this background, we proposed that these two anthropometric measures, in the selected women’s group, must gain some specific characteristics that are different from criteria provided by the conventional definition, used for analysis. If it is true, these characteristics may be also used for recognition of women at high CV risk.

We found, for women with MS, the waist circumference threshold of 89 cm (Table 6) (Fig. 4, right), that is much above the criterium of 80 cm of the IDF definition, used for analysis, indicating predisposition of these women for abdominal fat accumulation. This predisposition may be due to the effect of both, hypertension and menopause, on abdominal fat accumulation [34, 72]. This observation is supported with the result that also women with isolated hypertension had waist circumference values that are above the standard criterium of 80 cm (84.74 ± 5.28) (Table 4) (Fig. 4, right). When increased body weight is added to these two factors, they all may act synergistically on abdominal fat accumulation, worsening further metabolic and CV status [35, 73]. This pathophysiology chain reaction can be used to explain our results that almost all women with MS had increased body weight (BMI > 25.5 kg/m^2^) (Table 6) (Fig. 4, middle). This BMI cut-of value, as based on its good statistical performances to separate women with from those without MS, can be even used as a freestanding rule for recognition of women with MS (Table 5) (Fig. 2, middle). Moreover, because the parameter BMI, but not the parameter waist circumference, showed significant association with MS in the LR model, the parameter BMI is likely to perform better, than the standard waist circumference measure, as a part of the MS definition (Table 7). Generalisability of this result, although obtained on a small size sample, can be achieved by its comparison with the results of other studies, where increased BMI, in hypertensive menopausal women, was showed to associate better with subclinical organ damage, than components of MS [74].

Only two parameters of those indicating conventional components of MS, triglycerides and fasting blood glucose, showed significant associations with MS in the LR model (Table 7). Also their cut-off values (of 1.7 and 5.7 mmol/L, respectively) were found congruent with the standard MS criteria, implicating good diagnostic compliance with the examined population group (Table 6). However, when the ability of their cut-off values to discriminate between women with and without MS is considered, then only the parameter triglycerides, but not the parameter fasting blood glucose, can be used as a freestanding MS diagnostic tool for recognition of women with MS (Table 6) (Fig. 4, left). Our results provide even more details, indicating that information on increased serum triglycerides (above the cut-off value for MS) can be used with the high accuracy (of 96.8%) to identify around a half (62/131) of women with MS (DT rules, Fig. 6). Furthermore, based on a high degree of overlap between MS and diabetes, found for women in this sample (53/55), this information can also serve as a screening tool for women at high CV risk. There are pieces of evidence to support this assumption, showing that serum triglycerides are more markedly expressed when MS and diabetes are superimposed to each other, than when either of them stands alone [28, 75]. Distinctly from the parameter triglycerides, the parameter fasting blood glucose does not seem appropriate as a single marker of MS in this selected women’s group, because its cut-off value of 5.7 mmol/L failed to accurately classify a large part of women in the sample (Table 6). Explanation for this failure may be found in the fact that a large portion of women with MS have already had a diagnosis of diabetes. Another argument may be a piece of evidence indicating that impaired glucose tolerance, in women, in contrast to men, beter complies with impaired postload than fasting blood glucose, arguing for parameters changes in MS definition [43].

With respect to another conventional component of MS, HDL-cholesterol, our results suggest that the parameter total serum cholesterol and its cut-off value of 6.0 mmol/L perform better as a component of MS, than the parameter HDL-cholesterol. This conclusion is based on the good ability of this cut-off value to recognise women with MS (Tables 4 and 6) and the results of LR modeling, where the parameter total serum cholesterol, but not the parameter HDL-cholesterol, showed significant association with MS (Table 7). Increased total serum cholesterol can be considered the specific characteristic of hypertensive menopausal women, because both factors, hypertension and menopause, were found to increase total serum cholesterol [35]. As our results also suggest, even more favorable marker of MS, than increased total serum cholesterol, might be information on using cholesterol lowering drug statins. High diagnostic accuracy (of 89.4%) of this information to identify a large part of women with MS (66/131) (Fig. 7), is comparative to that on increased triglycerides (62/131) (accuracy 96.8%) (Fig. 6). This information must be, however, used with a caution, because its operative value may depend on how strictly prescription rules for statins are used by family doctors in a local environment. According to the guidelines, statins are prescribed either to diabetics or non diabetics with high serum total cholesterol; in both cases, this information indicates patients at high CV risk [28].

When these results on the diagnostic accuracy of the conventional components of MS, in the selected group of hypertensive menopausal women, are taken together, we can conclude that the best markers of MS, used either separately or as a combination, include: BMI > 25.5 kg/m^2^, increased triglycerides > 1.7 mmol/L and increased total serum cholesterol > 6.0 mmol/L, or information on statins use. For a smaller part of hypertensive menopausal women, for which either of these information does not provide the meaningful framework for MS diagnosis, diagnosis of diabetes, or rules based on a mixture of parameters, indicating also comorbidities and socio-behavioural factors, can provide the reasonable means (Figs. 6 and 7).

### Comorbidities, socio-demographic and lifestyle factors associated with MS

According to the above discussion and when the overall predictive accuracy of developed LR models is considered (Tables 7, 8, 9 and 10), it allows for a conclusion that the conventional components of MS and related metabolic factors are the best predictable means of MS. However, a full-range of the MS phenotype variability to be achieved, this will require also other factors to be used for predictive modeling. The example is when a range of laboratory parameters showed significant differences between women with and without MS (Table 4) and when many of these parameters were selected in the LR model, contributing to the model’s predictive power, along with the conventional components of MS (Table 7). Yet a range of laboratory parameters, taken as a whole, but not any of them, if taken as an alone, allow for pathophysiology disorders to be recognised, for which also other sources of information provide evidence for their associations with MS. Pathophysiology disorders, indicated with these results, include: renal function decline, chronic inflammation and disturbed haemorrheology [38, 39, 76]. We propose that a range of laboratory parameters that are associated with MS can vary in some degrees in different population groups, according to characteristics of patients in the sample and the availability of parameters, although it is not expected to go out of the boundaries of the panel of data that are indicated in this study.

When interpreted in this context, then the parameters monocytes% (Mo) and leukocyte count (Le), that were found significant in the LR model (Table 7), but not in the analysis of differences (Table 4), can be viewed as a part of the common inflammation/disturbed haemorrheology disorder, for which the parameter Htc, indicating increased haematocrit values, yet represents a more general mean [76]. Namely, when a high specificity of the cut-off value of this parameter (Table 4) and its big OR obtained in the LR model (Table 7) are taken into account, that means that only this parameter, of all laboratory parameters examined, is worthy of consideration to be used as a single marker for MS diagnosis. Practical implication is that the haematocrit values above the threshold of 41%, if found in menopausal women with hypertension, could be considered as the MS diagnosis, without the need of having information on conventional components of MS.

As we expected, analysis of comorbidities has provided information that can be used to improve the phenotype profilling of hypertensive menopausal women with MS. As added value, this analysis has enabled some glimpses on mechanisms of MS generation, thus paving the way for future research.

Medical conditions, that had been selected in the first step selection, according to the analysis of differences, were those ones for which also evidence show their associations with MS, including: CVD (parameters CoHD and CHD), sleep disorders, anxiety/depression, cognitive disorders, psychotic disease and inflammatory bowel disease (Table 5) [40–42, 77, 78]. This agreement between the knowledge and our results argues towards the feasibility of the proposed research approach for MS assessment that is based on using data from GP eHRs and a smal size sample. More specific analysis of the second step, based on using LR modeling, showed a more restricted panel of medical conditions as associated with MS, including: CVD, inflammatory bowel disease, psychotic disorders and anxiety/depression (Table 8). Here, a caution must be declared. It is possible that inadequately determined frequencies of the diagnoses of sleep disorders, anxiety/depression and cognitive disorders, for which the ICD-10 coding system shows insufficient, especially when older population is considered, might have influenced their wrong selection into the LR model [79]. For the needs of phenotype profilling, a procedure that relies on a comprehensive analysis of all relevant medical conditions associated with MS, diagnoses of these conditions have to be more accurately determined. This would be routinelly possible, if the available scoring systems for detection of these disorders were included as a part of GP eHRs, ensuring a systematic approach to diagnosis.

Because inflammatory bowel disease and psychotic disease were presented with low frequency in this sample, practical usefulness can be considered for the diagnoses of CVD and anxiety/depression. Of these two, the potential for improving prevention of CVD, in menopausal women, can be considered for the diagnosis of anxiety/depression. This assumption is also supported with the results of the DT model, where this diagnosis was unveiled as a part of the rule for MS recognition, being placed in the same clinical context with the new onset diabetes (indicated with the category “non treated diabetes“) and a long-term hypertension (of more than 5 years of duration) (Fig. 7). That anxiety/depression might be a mechanism that in menopausal women drives development of MS and other CV factors, this is indicated, although indirectly, with the results of the LR modeling process, where comorbid disorders were put together with data indicating social factors and lifestyles (Table 8). Based on these results, a social context was identified that in menopausal women can favour MS development, including alcohol use behaviour (a mechanism of how women cut down their intrinsic tensions) and lower socio-economic status (known to produce chronic social stress and unhealthy behaviours, leading to increase in CV risks) [80, 81].

Another comorbid disorder, for which our results also indicate its association with MS, although more indirectly, is impaired renal function, represented with the parameter GFR. It is found as a non significant part of the LR model (Table 7) or as a hidden within the combined DT rules (Fig. 6). Low emphasis that it is put on this parameter, may be due to the low overall level of expression of this disorder in women in the sample, as a progression of this disorder is expected to occur in older age [53].

Although all medications that we used for analysis were also selected in the LR model, indicating that all of them can contribute to MS diagnosis, those ones that showed significant associations with MS were beta-blockers, metformin and statins (Table 9). As we have already stated for statins, information on using these medications can help family doctors recognise the specific women’s groups. In this terms, beta-blockers can indicate women diagnosed with CVD and metformin can indicate those diagnosed with a new onset diabetes [82]. Strong emphasis that in our study is put on association between the use of statins and MS, as based on both, results of the LR model and DT rules (Table 9) (Figs. 6 and 7), can be also reflective of their proposed influence on MS and diabetes development [83]. If proved true, this statement would have implications on changing the prescription rules, from the current “one-fit-all “to a more diversificated approach, that will be able to address, more specifically, narrowly defined patient groups, such as a group of menopausal women with hypertension.

Relationships between menopause, hypertension and diabetes duration and the time when MS does emerge, have become more reliable when presented graphically, than just analysed by the modeling. Namely, results of the LR model showed as the time when MS most intensively emerges the period of 1–3 years after menopause (corresponding with early postmenopause) (Table 10) (Fig. 2). On the bar graph, this period is represented with the big dysproportion in frequency of women with and without MS, indicating intensive transition, placed in the period of 1–3 years of menopause duration (Fig. 5, left). What else was possible to perceive from the graph, but that was not possible otherwise, is an overview of the MS frequency distribution througout the periods of menopause duration. This way, it looks like that the bundles of the MS frequency are devided into the two discrete periods: one less intensive (the option “No“), indicating time close to menopause and corresponding to late menopause transition, and the other more intensive (options 1–3 and > 3), corresponding with early postmenopause (Fig. 2) (Fig. 5, left). These two periods are also emphasised with evidence as critical for the emergency of MS [48]. This gives confidence to our research approach that is based on using a large dataset and a combination of analytical methods, to answer some complex questions.

A new and intruiging finding that arises from these results is related to our impression on the possible coincidence of a new onset diabetes and the emergency of MS. This impression is based on the results of the LR model (Table 10), where parameters “diabetes diagnosis “and “diabetes duration 0″, indicating recently developed diabetes, showed significant associations with MS (based on the big ORs). This impression have become even more reliable when results of the LR model were presented graphically (Fig. 5, right). On the bar graph, MS transition is placed into the category of a new onset diabetes (marked with“0″). These results are complementary to the high degree of overlap between MS and diabetes, found for women in the sample (53/55). This idea, on possible simultaneous development of MS and diabetes, as a specific trait of menopausal women with hypertension, is exciting from the preventive aspects and deserves further evaluation, especially because evidence on this issue are also limited. The only report that we found is that on a greater increase in CV risk through the appearance of diabetes, that is a characteristic of women with MS, in contrast to men [84]. Our results provide even more complete information on this issue, by placing the coincidence of MS and a new onset diabetes into a wider clinical context, characterised also with a long-term hypertension (of more than 5 years of duration) and anxiety/depression (Fig. 7) (Fig. 5, middle). This way, pieces of information, provided by different methods for data analysis, converge into a common, complex view.

### Practical protocol, for use in GP, for fast recognition and preventive management of menopausal women at high CV risk

The group of women in which CV risk factors are expected to intensivly emerge is in the age of 50–55 years and diagnosed with hypertension. If these women have incresed BMI, this very probably means the diagnosis of MS. Other relatively accurate single-parameter rules, to capture a prevalent part of women with MS, include: increased triglycerides, above 1.7 mmol/L, increased total serum cholesterol, above 6.0 mmol/L, and information on statins use. Frequent follow up of these women on a new onset diabetes is credible, because of the possible simultaneous onset of MS and diabetes. A special attention, in terms of prevention of CVD, should be also put on women with anxiety/depression and mild renal impairment. Women with a new onset diabetes should be provided with intensive treatment of CV risk factors, because of the expected high burden of CV risk factors in this population group.

## Conclusions

The added value of this study goes beyond the current paradigm on MS. Results indicate characteristics that can be used to improve the diagnosis of MS according to the narrowly defined specific population group such as menopausal women with hypertension. Although components close to the conventional definition of MS bear the most of the diagnostic capacity for MS, to capture the full-range variability of the phenotypes, a mixture of factors, including also comorbidities and other clinical and socio-behavioural factors, should be used into consideration. Advantages would be in GP, for improving prevention of CVD in women, especially because the current methods for CV risk estimation, for this specific population group, show insufficent.

To enable the routine use of data from GP eHRs for this kind of research, the panel of data that are systematically recorded should include some other parameters, in addition to the usual structured data.

These necessary additional data are information on socio-demographic and lifestyle factors and scoring systems for diagnosing medical conditions for which the standard coded diagnosis system shows limited, such as anxiety/depression and sleep and cognitive disorders. What is also important, is to achieve harmonisation, among family doctors, in diagnosis and prescription rules, mostly related to the diagnoses of diabetes and anxiety/depression and the statins prescription. The challenging issue will be also training of general practitioners in skills for multiple results integration and their harmonisation with knowledge.

Several new findings, specifically associated with the characteristics of the examined population group, have arised from this study and require further elaboration, for their possible practical implications. These findings include: the existence of the two main lipid disorders represented with increased triglycerides and total serum cholesterol; the possible involvement of statins in the pathophysiology of MS and diabetes development; the possible coincidental development of diabetes and MS; the preventive potential, for the development of MS and diabetes, of recognition of anxiety/depression in menopausal women with a long-lasting hypertension.

## Abbreviations

CV: Cardiovascular
DWBC: White blood cell differential
eHRs: Electronic health records
FGlu: Fasting glucose
GFR: Glomerular filtration rate
GP: General practice
HDL: High density lipoprotein
ICD-10: International statistical classification of diseases and related health problems 10th revision
IDF: International diabetes federation
LDL: Low density lipoprotein
MS: Metabolic syndrome
TG: Triglycerides
